# Retraction Note: Dose selection of central or peripheral administration of sufentanil affect opioid induced cough?: a prospective, randomized, controlled trial

**DOI:** 10.1186/s12871-024-02522-9

**Published:** 2024-04-12

**Authors:** Jiabei He, Ling Zhu, Huichen Zhu, Xinyu Gu, Peiying Li, Yuting Yang, Liqun Yang

**Affiliations:** Department of Anesthesiology, Renji Hospital, School of Medicine, Shanghai Jiaotong University, Shanghai, 200127 China

**Retraction Note: BMC Anesthesiol 18, 38 (2018)**

**https://doi.org/10.1186/s12871-018-0502-z**

The authors have retracted this article because, after publication, concerns were raised regarding the statistics used to analyze the data. The authors found that they had applied parametric tests to the analysis of the primary outcome, the data ‘occurrence of coughs’ instead of a chi-square test. Therefore, the data and calculations do not support the conclusion.

The authors sincerely apologize to the scientific community for any confusion caused by these errors and are preparing a new manuscript for submission for peer review.

All authors agree with this retraction.

